# Antimicrobial Susceptibility and Resistance Mechanisms in *Mannheimia haemolytica* Isolates from Sheep at Slaughter

**DOI:** 10.3390/ani13121991

**Published:** 2023-06-14

**Authors:** Julio Alvarez, Johan M. Calderón Bernal, Laura Torre-Fuentes, Marta Hernández, Chris E. Pinto Jimenez, Lucas Domínguez, José F. Fernández-Garayzábal, Ana I. Vela, Dolores Cid

**Affiliations:** 1 Departamento de Sanidad Animal, Facultad de Veterinaria, Universidad Complutense, 28040 Madrid, Spain; jalvarez@visavet.ucm.es (J.A.); johancal@ucm.es (J.M.C.B.); lucasdo@ucm.es (L.D.);; 2 Centro de Vigilancia Sanitaria Veterinaria (VISAVET), Universidad Complutense, 28040 Madrid, Spain; ltorre04@ucm.es; 3 Laboratorio de Biología Molecular y Microbiología, Instituto Tecnológico Agrario de Castilla y León, 47071 Valladolid, Spain; hernandez.marta@gmail.com; 4 London School of Hygiene and Tropical Medicine, University of London, London WC1E 7HT, UK; 5 Facultad de Medicina Veterinaria, Universidad Nacional Mayor de San Marcos, Lima 15021, Peru

**Keywords:** *Mannheimia haemolytica*, antimicrobial resistance, mobile genetic elements, *tetH*, *strA*, *sul2*, sheep

## Abstract

**Simple Summary:**

*Mannheimia haemolytica* is a key bacterial pathogen contributing to important ruminant diseases and accounting for a large proportion of overall antimicrobial use in cattle and sheep. The recent emergence of ovine strains with reduced susceptibility to antimicrobials—which could lead to treatment failure, increased costs of livestock production, and the dissemination of antimicrobial-resistant genes to other bacteria and possibly the environment—is of concern. This study investigated the levels of antimicrobial resistance of *M. haemolytica* isolated from clinically healthy sheep at slaughter and the genetic bacterial resistance mechanisms. Low levels of phenotypic resistance were detected for most of the antimicrobials tested except for tetracycline (4.3%) and tylosin (89.1%). A few antimicrobial resistance determinants were found in the genome of six out of nine isolates, consisting of genes conferring resistance to tetracyclines (*tetH)*, aminoglycosides (*strA*), and sulfonamides (*sul2*), which were sometimes linked to the presence of plasmids but did not always lead to resistance phenotypes. Our results suggest that there is limited resistance in *M. haemolytica* strains of veterinary origin, but the presence of several resistance genes, some of which were found in mobile genetic elements that play a major role in the dissemination of antimicrobial resistance in members of the *Pasteurellaceae* family, deserves further consideration.

**Abstract:**

*Mannheimia haemolytica* is the main pathogen contributing to pneumonic pasteurellosis in sheep. The aim of this study was to investigate the antimicrobial resistance levels in *M. haemolytica* isolates from the lungs of slaughtered sheep and to examine the genetic resistance mechanisms involved. A total of 256 *M. haemolytica* isolates, 169 from lungs with pneumonic lesions and 87 from lungs without lesions, were analyzed by the disk diffusion method for 12 antimicrobials, and the whole genome of 14 isolates was sequenced to identify antimicrobial resistance determinants. Levels of phenotypic resistance ranged from <2% for 10 antimicrobials (amoxicillin, amoxicillin-clavulanic, ceftiofur, cefquinome, lincomycin/spectinomycin, gentamicin, erythromycin, florfenicol, enrofloxacin, and doxycycline) to 4.3% for tetracycline and 89.1% for tylosin. Six isolates carried *tetH* genes and four isolates carried, in addition, the *strA* and *sul2* genes in putative plasmid sequences. No mutations associated with macrolide resistance were identified in 23 rDNA sequences, suggesting that the *M. haemolytica* phenotypic results for tylosin should be interpreted with care in the absence of well-established epidemiological and clinical breakpoints. The identification of strains phenotypically resistant to tetracycline and of several resistance genes, some of which were present in plasmids, highlights the need for continuous monitoring of susceptibility patterns in *Pasteurellaceae* isolates from livestock.

## 1. Introduction

*Mannheimia haemolytica* is an important ruminant pathogen contributing to bovine respiratory disease (BRD) in cattle and pneumonic pasteurellosis in sheep and accounting for a large proportion of overall antimicrobial use in both cattle and sheep [1]. *M. haemolytica* is a commensal in the nasopharynx that can lead to clinical disease usually when animals are exposed to predisposing factors, which include stress induced by changes in the environment or by other bacterial and viral infections [2]. In sheep, pneumonic pasteurellosis can present as acute disease, but subclinical or chronic cases are frequent, and bronchopneumonia lesions linked to these presentations are common findings at the slaughterhouse [3]. *M. haemolytica* can be isolated from pneumonic lungs and from the lungs of healthy sheep, with isolates retrieved from both types of clinical presentations showing a similar genetic background consistent with the view that individual cases of pneumonia are due to commensal *M. haemolytica* strains reaching the lungs from the upper respiratory tract in the presence of predisposing factors [3]. Optimized control strategies for pneumonic pasteurellosis in sheep require information about the antimicrobial susceptibility of *M. haemolytica* respiratory isolates. However, only a limited number of studies have evaluated the in vitro antimicrobial susceptibility of ovine *M. haemolytica* isolates, and most of these included a limited number of strains [4,5]. Although most clinical ovine isolates are usually phenotypically susceptible to antibiotics, the emergence of ovine strains with reduced susceptibility to antimicrobials has been reported [4,5]. These potentially resistant bacteria may lead to treatment failure, increased costs of livestock production, and the dissemination of antimicrobial-resistant genes to other bacteria and possibly the environment [6]. Nevertheless, due to the absence of clinical efficacy studies on the dosing and route of administration of antimicrobial agents used in veterinary medicine to treat animal diseases for multiple pathogen–drug combinations, the clinical significance of in vitro resistance (or reduced susceptibility) in relation to the risk of therapeutic failure is difficult to ascertain [7]. *M. haemolytica* associated with bovine respiratory disease (BRD) harboring multiresistance-mediating integrative and conjugative elements (ICEs) has been identified in North America and in European countries [6,8]. However, despite the noteworthy emergence of antimicrobial resistance in *M. haemolytica* isolates from sheep, very little knowledge exists about the genetic mechanisms conferring resistance. In this context, it is necessary to generate more information regarding the frequency of antimicrobial resistance in *M. haemolytica* isolates originating from different livestock animals and countries [1]. Thus, the current study aimed to investigate the levels of antimicrobial resistance of *M. haemolytica* isolated from the lungs of slaughtered sheep in Spain over a period of 22 months and to examine the underlying genetic resistance mechanisms.

## 2. Materials and Methods

### 2.1. M. haemolytica Isolates 

A total of 256 *M. haemolytica* isolates were included in this study. Isolates were retrieved from lung samples collected at slaughter from 139 apparently healthy lambs based on the absence of any clinical sign of disease, external lesions, weakness, or stress during the compulsory antemortem examination performed less than 24 h before slaughter. Of these isolates, 169 were recovered from lungs with pneumonic lesions (*n* = 92 sheep with pneumonic lungs) while 87 were isolates obtained from lungs without pneumonic lesions (*n* = 47 animals with non-pneumonic lungs). Pneumonic lesions were defined as the presence of clearly demarcated areas of consolidation in cranioventral lung lobules [3]. Between one and five isolates per lung (i.e., per animal) were included (one isolate from 56 lungs, two isolates from 55 lungs, three isolates from 23 lungs, four from four lungs, and five isolates from one lung). During a period of 22 months, lung samples were collected at slaughter in three abattoirs that received animals from seven farms located in three Spanish provinces [3]. Lung samples were cultured on Columbia blood agar plates (bioMérieux), which were incubated at 37 °C for 24 h. Isolates were biochemically identified using the commercial identification system Diatabs Diagnostic Tablets (Rosco Diagnostica, Taastrup, Denmark), as previously described [9]. Biochemical identification was further confirmed by a species-specific polymerase chain reaction (PCR) assay [10]. After identification, bacterial isolates were frozen and stored at −80 °C until use.

### 2.2. Antimicrobial Susceptibility Testing 

The resistance phenotype of all 256 *M. haemolytica* isolates to 12 antimicrobials was investigated using the disk diffusion method as described in the Clinical and Laboratory Standards Institute document M31-A3 [11] using Mueller–Hinton plates (Oxoid, Ltd.; Madrid, Spain). Inocula were prepared from a 24-h Columbia blood agar plate by resuspending four colonies in 5 mL of Mueller–Hinton broth and adjusting to a 0.5 McFarland standard. The following commercial antimicrobial disks (A/S Rosco Diagnostica, Taastrup, Denmark, UK) were used: amoxicillin (AMOX, 30 μg), amoxicillin/clavulanic acid (AMC, 30/15 μg), ceftiofur (XNL, 30 μg), cefquinome (CFQUI, 30 μg), lincomycin/spectinomycin (LI+SP, 15 + 200 μg), gentamicin (GEN, 10 μg), erythromycin (ERY, 78 μg), tylosin (TYLO, 150 μg), florfenicol (FFC, 30 μg), enrofloxacin (ENRO, 10 μg), doxycycline (DOXYC, 80 μg), and tetracycline (TET, 30 μg). The agar plates were examined after 24 h of incubation at 37 °C. Breakpoints used for the qualitative interpretation of results and classification of isolates into wild-type (susceptible) and non-wild-type (resistant) categories for CEFT and FFC were those recommended by the Clinical and Laboratory Standards Institute (CLSI) for *M. haemolytica* [12], while for GEN and TET the breakpoints were those recommended by the CLSI for bacteria isolated from animals [13]. For antimicrobials with no CLSI-recommended breakpoints (AMOX, AMC, CEFQ, LI+SP, ERY, TYLO and ENRO, DOXYC), those recommended by Rosco Diagnostica [14] were used (Table 1). *Staphylococcus aureus* ATCC 25923 and *Escherichia coli* ATCC 25922 were included as quality controls with each batch of organisms tested. Multidrug resistance (MDR) was defined as acquired non-susceptibility to at least one agent in three or more antimicrobial categories. A bacterial isolate was considered resistant to an antimicrobial category when it was resistant to at least one agent in that category. The resistance phenotype of a selection of six isolates that yielded inhibition zone diameters (IZDs) for TYLO ranging from 10 to 21 mm and subjected to whole-genome sequencing (see below) was further investigated using the microdilution method to assess whether the presence of antimicrobial resistance markers detected through sequencing translated into differences in the minimal inhibitory concentrations (MICs) for a range of antimicrobials. Briefly, isolates were inoculated into Mueller–Hinton broth supplemented with 3% lysed horse blood and their MICs for the antibiotics included in panel EUVSEC3 were determined using the twofold broth microdilution reference method according to ISO 20776-1:2021. 

### 2.3. Whole-Genome Sequencing (WGS) 

The whole genomes of nine isolates representative of those phenotypically resistant to TYLO (with IZDs ranging from 10 to 22 mm) and five isolates representative of those phenotypically susceptible to TYLO (with IZDs ranging from 23 to 30 mm) based on the disk diffusion method (see Results) were sequenced using Illumina technology to identify any resistance mechanisms associated with their resistance phenotype. DNA was purified from axenic cultures with the Qiagen DNA Blood & Tissue Kit, following the manufacturer’s instructions, and quantification of the DNA concentration and further libraries was made using a Qubit^®^ fluorometer (Invitrogen; Waltham, MA, USA). WGS libraries were prepared from 1 ng of bacterial DNA using the Nextera XT DNA Library Preparation Kit. The concentrations of each library were adjusted to 4 nM in order to obtain equimolar DNA concentrations in a single pool of libraries to be sequenced in a MiSeq device (Illumina; San Diego, CA, USA).

Illumina reads were processed using in-house pipelines [15,16]. Briefly, adaptors in the raw reads were removed and low-quality raw reads were filtered out with Trimmomatic. The reads that passed the quality control with FastQC were assembled by SPAdes and the quality of the assemblies was evaluated with QUAST. Assemblies were then screened for the presence of antimicrobial resistance genes using Resfinder with default parameters and for the presence of plasmid replicons with PlasmidFinder using an identity threshold of >80%. Plasmid identities were further confirmed with MOB-suite [17]. The presence of additional resistance markers (*macA* and *macB* genes [18] *bla_ROB-2_* gene [19]) was investigated in the annotated assemblies using PROKKA and by aligning the raw reads from the isolates to the sequence of the *bla_ROB-2_* gene [19], respectively.

Finally, in order to identify point mutations associated with resistance to macrolides (23S rDNA sequence) [20] and fluoroquinolones (quinolone resistance determinant regions, QRDR) [21], reads were aligned to the reference *M. haemolytica* genome CP005383.1 using bwa with defaults parameters. The resulting SAM files were sorted and compressed into BAM files using SAMtools. Variant calling was performed by BCFtools using the ‘mpileup’ and ‘call’ command options, excluding SNPs with a base quality lower than 30 and a mapping quality lower than 30. Then, consensus sequences for each strain were created using the BCFtools ‘consensus’ command. The six copies of the 23S rRNA subunit were located in the annotation of the reference genome, and the consensus sequences of each strain were annotated with PROKKA in order to locate the 23S rRNA operons for each strain. The 23S operons from all strains were then extracted and aligned to the 23S reference sequence NR103087.1. Variant calling was performed for each operon of each isolate as described above. SNPs were outputted in vcf files to identify the presence of mutations associated with macrolide resistance in the 23S rRNA. The raw reads generated in this study were deposited in the European Nucleotide Archive under project PRJEB61140.

### 2.4. Statistical Analysis

The association between the clinical origin of the isolates and their antimicrobial susceptibility was determined using the chi-square test, with *p* < 0.05 considered significant. In order to account for the lack of independence between isolates coming from the same animal (i.e., lung), the significance of the association between the presence of lesions and the antimicrobial susceptibility to a given antimicrobial (or their resistotype) was also tested using a mixed logistic regression model including the animal as a random effect. Data were analyzed using the Epi InfoTM 7.2.5 software of the Centers for Disease Control and Prevention (CDC; https://www.cdc.gov/epiinfo/esp/es_pc.html (accessed on 13 April 2023)).

## 3. Results

### 3.1. Phenotypic Susceptibility to 12 Antimicrobials

The results of in vitro susceptibility testing by the disk diffusion method for the 256 ovine *M. haemolytica* isolates are shown in Table 1. Using the breakpoints indicated in the table, less than 2% of the isolates were classified as resistant for 10 of the 12 antimicrobials tested, ranging from 0% for LI+SP, ERY, and FFC to 1.2% for AMOX and CEFQ (Table 1). A higher level of resistance was detected for TET (4.3%), and the highest level was found for TYLO (89.1%; Table 1). No statistically significant differences (*p* < 0.05) were detected between the proportions of resistant isolates in pneumonic and non-pneumonic lungs for all antimicrobials tested (Table 1). In this study, 11 resistotypes were identified, with most of the isolates (83.6%) being resistant only to the macrolide TYLO, whereas 9.8% of the isolates were susceptible to all antimicrobials (Table 2). No significant differences in the resistotype frequencies between isolates from lungs with and without pneumonic lesions were observed.

### 3.2. WGS Analysis

Three isolates with IZD values above and two isolates with IZD values below the TYLO breakpoint were discarded due to the low quality of the reads. Among the remaining 10 isolates (six and four with IZDs above and below the selected TYLO breakpoint, respectively), antimicrobial resistance determinants were found in six: two carried genes conferring resistance to TETs (*tetH*), aminoglycosides (*strA*), and sulfonamides (*sul2*), while the remaining four isolates all carried the *tetH* gene (Table 3). Putative plasmid sequences were found in five strains (plasmid replicons rep21 and IncP according to PlasmidFinder, present in three different strains, and a putative plasmid sequence in two strains found by MOB-suite) (Table 3). The sequences identified as plasmid-associated by MOB-suite, of 5488 bp, found in two different strains were identical and also carried the *strA* and *sul2* genes. These putative plasmids were very similar (>99.9% identity) to plasmid pHN06 (5360 bp), found in a *Pasteurella multocida* strain retrieved from a pig with atrophic rhinitis in China that also harbored AMR genes *strA* and *sul2* [22]. No mutations previously associated with resistance to fluoroquinolones or macrolides were identified in the QRDR and 23 rDNA sequences, respectively, from any of the sequenced strains.

The MIC values, available in five of the six sequenced strains with IZD values above the TYLO breakpoint, were identical for 10 of the 15 antimicrobials tested, with values differing by more than one dilution for sulfamethoxazole, amikacin, trimethoprim, TET, and GEN (Table 3).

Epidemiological cut-off values (ECOFFs) within the concentration range included in the EUVSEC3 plate were only available for TET; based on the ECOFF, two isolates (both carrying the *tetH* gene) were classified as resistant, whereas the remaining four isolates (one of which also carried the *tetH* gene) were considered susceptible (Table 3). For the remaining four antimicrobials in which different MICs were found, higher values were not associated with the carriage of specific resistance genes (Table 3).

## 4. Discussion

*M. haemolytica* is a ruminant-specific pathogen associated with pneumonic pasteurellosis in sheep, a relevant disease due to the significant economic losses that it causes [2]. It is generally accepted that the mere presence of *M. haemolytica* in lungs is not sufficient to produce lesions [2] and that individual cases of pneumonia are associated with commensal *M. haemolytica* strains of the upper respiratory tract that reach the lungs due to predisposing factors [3]. Antimicrobial surveillance provides important information that is necessary for epidemiological knowledge and empirical treatment options. In this study, we investigated the antimicrobial susceptibility of 256 *M. haemolytica* isolates recovered at slaughter from ovine lungs with and without pneumonic lesions. Moreover, we also determined the genetic antimicrobial mechanisms of resistance in a selection of *M. haemolytica* resistant to macrolides.

Overall, most ovine *M. haemolytica* isolates investigated in this study were classified as susceptible to the majority of (10 of 12) antimicrobials tested, with resistance rates between 0% and 1.2% (Table 2). These results agree with the high susceptibility of ovine *M. haemolytica* isolates to most antimicrobials observed in different studies [4,5]. Despite this overall high susceptibility to most antimicrobials, moderate resistance rates were observed for TET (Table 1). TETs are among the most frequently used antimicrobials in animals [23]. Therefore, these resistance rates may reflect the predominance of their use in sheep farming. The *tetH* TET-resistance gene, encoding an energy-dependent membrane-associated protein that exports TETs out of the cell [24], was found in six of the 10 sequenced strains, of which only two were resistant according to the disk diffusion test. When considering the results from the microdilution test (only available for six of the 10 sequenced strains), two of the three strains carrying the gene were classified as resistant (Table 3). This gene was identified as the predominant *tet* gene in *Pasteurellaceae* isolates of bovine and swine origin in North America in the 1990s [24] and was more recently described in phenotypically resistant *M. haemolytica* isolates retrieved from cattle in the United States [25]. It was also the most common resistance gene found using metagenomics in nasopharyngeal samples from chronically ill feedlot cattle in Canada [26], suggesting it may play a significant role in the occurrence of TET resistance in respiratory pathogens from ruminants. However, the presence of *tetH* in *M. haemolytica* isolates classified as phenotypically susceptible to certain TET (chlortetracycline) based on clinical breakpoints, as found in this study, has also been previously described, further highlighting the complexities of predicting resistance phenotypes from genetic data in this bacterial species [27].

The most striking result in this study was the very high resistance rate to TYLO based on the breakpoint used (89.1%; Table 1). This antimicrobial has been classified by the World Health Organization as critically important with the highest priority for human medicine [28], and therefore the spread of genetic determinants mediating this antimicrobial may represent a serious concern. Similar levels of TYLO resistance have been observed in other respiratory pathogens such as *Pasteurella multocida* in sheep and in pigs [29,30] in Spain. It has been suggested that the low degree of in vitro susceptibility of these bacteria to this macrolide could be due to the long-term exposure to this agent, given that it was used at subtherapeutic doses as a growth promotor until its ban in 1999 in Spain [31]. The acquired resistance to macrolides in members of the family *Pasteurellaceae* has been associated with the presence of the macrolide resistance gene coding for an efflux pump (*msrE*), genes that cause methylation of the ribosomal target (*ermA, ermC, and erm42*), and the gene that codes for a phosphorylase-inactivating enzyme (*mphE*) [31]. In addition, rRNA mutations that confer resistance to macrolides have been described in field isolates of *M. haemolytica* and *P. multocida* in cattle [20] and of *Haemophilus parasuis* in pigs [31]. However, no genetic determinants (antimicrobial resistance genes or point mutations) conferring resistance to macrolides were identified in any of the isolates sequenced in this study, regardless of their TYLO resistance phenotype (Table 3). Furthermore, no difference has been reported in treatment efficacy when using TYLO or tulathromycin in cattle from which TYLO-resistant (and tulathromycin-susceptible) *Pasteurellaceae* isolates had been retrieved, indicating a lack of direct correlation between in vitro resistance and therapeutic success [32]. Previous studies have also reported very high MIC values in *M. haemolytica* isolates retrieved from goats [33] and cattle [34] while also being predominantly susceptible to other macrolides (tulathromycin, tilmicosin) and/or not harboring known genetic determinants conferring resistance mechanisms to this antimicrobial class. Altogether, these findings suggest that *M. haemolytica* antimicrobial susceptibility test results for TYLO should be interpreted with care in the absence of well-established epidemiological and clinical breakpoints, since the lack of a correlation between genetic and phenotypic resistance data observed here and elsewhere could be due to the presence of as-yet unrecognized resistance mechanisms but also due to inappropriate breakpoints [27].

Mobile genetic elements are known to play a major role in the dissemination of antimicrobial resistance in members of the *Pasteurellaceae* family [6]. In this study, we identified the sequence of a short (~5300 bp) plasmid carrying both *sul2* and *strA* resistance genes (Table 3). The co-occurrence of these two genes in plasmids carried by clinical *asteurellaceae* strains has already been described in isolates retrieved from cattle and swine in several countries [35,36] including Spain [37]. The plasmid retrieved here had a high level of identity with a plasmid sequence originally described in a *P. multocida* strain from a diseased pig in China [22]. Even though the presence of the *strA* and *sul2* genes was not associated with increased MIC values to the sulfonamides and aminoglycosides included in the antimicrobial susceptibility testing in this study, additional research is needed to assess their possible role in conferring resistance to members of this antimicrobial family.

## 5. Conclusions

In conclusion, we found a very limited proportion of resistance to all antimicrobials except TYLO in *M. haemolytica* isolates retrieved from clinically healthy animals sampled at slaughter. The high levels of TYLO-resistant isolates based on the breakpoint used, however, should be interpreted with care, since no resistance mechanisms associated with macrolide resistance were found in a subset of sequenced isolates and very high MICs for this antimicrobial have been described in clinical *M. haemolytica* strains from ruminants, thus suggesting that the cut-off used here may not be differentiating truly non-wild-type genotypes. Our results agree with most evidence indicating a limited degree of in vitro resistance in *M. haemolytica* strains of veterinary origin; however, the identification of a subset of strains resistant to TET and of the presence of several resistance genes, some of which were carried in a plasmid, further highlights the need for continuous monitoring of susceptibility patterns in *Pasteurellaceae* isolates from livestock in order to detect newly emerging resistance mechanisms of clinical significance. The apparent lack of agreement between results from the predicted (based on the presence of AMR determinants) and the observed phenotypic resistance profiles stresses the need for additional data to calibrate WGS-derived data for in silico assessment of the susceptibility/resistance in clinical strains from *M. haemolytica*.

## Figures and Tables

**Table 1:** In vitro susceptibility by disk diffusion method for the 256 ovine *M. haemolytica* isolates.

Antimicrobial Agent	Units	IZD Breakpoints (mm)	No. of Isolates with IZD (mm) of: ^a^																Resistant Isolates (%) ^e^		
			7–8	9–10	11–12	13–14	15–16	17–18	19–20	21–22	23–24	25–26	27–28	29–30	31–32	33–34	35–36	>36	From Lungs with Pneumonic Lesions	From Lungs without Pneumonic Lesions	Total
AMOX	30 μg	≤16 ^b^	1		2					3	8	14	15	41	73	39	38	22	0.6	2.3	1.2
AMC	30/15 μg	≤16 ^b^			1			2			8	10	14	51	59	48	40	23	0.6	0.0	0.4
CEFT	30 μg	≤17 ^c^	1			1			3	1	5	6	9	35	57	39	37	62	1.2	0.0	0.8
CEFQ	30 U	≤19 ^b^	2				1				7	18	18	46	54	36	38	36	1.2	11	1.2
LI+SP	15/200 μg	≤16 ^b^							9	12	54	77	67	19	10	2	2	4	0.0	00	0.0
GEN	10 μg	≤12 ^d^	1			10	54	101	73	3	2	2	5	1	4				0.6	00	0.4
ERY	78 μg	≤18 ^b^							22	32	62	65	45	16	8	2	3	1	0.0	00	0.0
TYLO	150 μg	≤22 ^b^		1		3	8	32	117	67	20	1	3	1	1		1	1	87.6	91.9	89.1
FFC	30 μg	≤14 ^c^							1		5	11	20	50	64	42	49	14	0.0	00	0.0
ENRO	10 μg	≤16 ^b^				1	1				5	16	17	54	63	31	34	34	0.0	23	0.8
DOXYC	80 μg	≤18 ^b^						1	4	3	1	17	19	57	80	38	24	12	0.6	0.0	0.4
TET	30 μg	≤14 ^d^	5	3		3	3		4	4	36	72	60	43	16	3	2	2	5.9	1.1	4.3

Abbreviations: AMOX, amoxicillin; AMC, amoxicillin/clavulanic acid; CEFT, ceftiofur; CEFQ, cefquinome; LI+SP, lincomycin/spectinomycin; GEN, gentamicin; ERY, erythromycin; TYLOS, tylosin; FFC, florfenicol; ENRO, enrofloxacin; DOXYC, doxycycline and TET, tetracycline. ^a^ In order to present clearer data, inhibition zone diameters (IZD) have been grouped into 2-mm intervals. ^b^ Breakpoints recommended by Rosco Diagnostica (Neo-Sensitabs user’s guide; A/S Rosco Diagnostica) [14]. ^c^ Breakpoints recommended by the Clinical and Laboratory Standards Institute (CLSI) for *M. haemolytica* [12]. ^d^ Breakpoints recommended by the CLSI for bacteria isolated from animals [13]. ^e^ No statistically significant differences were detected between lungs with and without pneumonic lesions.

**Table 2:** Antimicrobial resistance patterns (resistotypes) and clinical origin of the *M. haemolytica* isolates.

Resistotype ^a^	Antimicrobial Family ^b^					No. (%) of *M. haemolytica* Isolates from Lungs			
						With Pneumonic Lesions	Without Pneumonic Lesions	Total	*p*
	MAC	LAC	TET	QUI	AMI	*n* = 169	*n* = 87	*n* = 256	
TIL						139 (82.2)	75 (86.2)	214 (83.6)	>0.05
TIL. TET						6 (3.6)	1 (1.1)	7 (2.7)	>0.05
TIL. AMOX						0	2 (2.3)	2 (0.8)	>0.05
TIL. CEFQ						0	1 (1.1)	1 (0.4)	>0.05
TIL. AMOX. AMC. CEFT						1 (0.6)	0	1 (0.4)	>0.05
**TIL. CEFQ. TET ^c^**						1 (0.6)	0	1 (0.4)	>0.05
TIL. TET. DOX						1 (0.6)	0	1 (0.4)	>0.05
TIL. ENRO						0	1 (1.1)	1 (0.4)	>0.05
ENRO.						0	1 (1.1)	1 (0.4)	>0.05
**CEFT. CEFQ. TET. GEN ^c^**						1 (0.6)	0	1 (0.4)	>0.05
TET						1 (0.6)	0	1 (0.4)	>0.05
None						19 (11.2)	6 (6.7)	25 (9.8)	>0.05

^a^ Abbreviations: AMOX, amoxicillin; AMC, amoxicillin/clavulanic acid; CEFT, ceftiofur; CEFQ, cefquinome; LI+SP, lincomycin/spectinomycin; GEN, gentamicin; ERY, erythromycin; TYLOS, tylosin; FFC, florfenicol; ENRO, enrofloxacin; DOXYC, doxycycline; TET, tetracycline; ^b^ MAC, macrolides; LAC, β-lactams; QUI, quinolones; AMI, aminoglycosides. ^c^ Multidrug-resistance (MDR) resistotypes, defined as acquired non-susceptibility to at least one agent in three or more antimicrobial categories, are highlighted in bold.

**Table 3:** Presence of resistance genes, plasmid sequences, and resistance profile according to the microdilution method in a subset of isolates.

		Resistance Genes			Plasmids			Antimicrobial Susceptibility Phenotype (Concentration Ranges Tested in μg/mL) ^a^														
ID	IZD-TYLO (mm)	*tetH*	*strA*	*sul2*	rep21	IncP	pHN06	SMX (8–512)	AMI (4–128)	TMP (0.25–16)	CIP (0.015–8)	TET (2–32)	MERO (0.03–16)	AZI (2–64)	NAL (4–64)	CHL (8–64)	FOT (0.25–4)	TGC (0.25–8)	TAZ (0.25–8)	COL (1–16)	AMP (1–32)	GEN (0.5–16)
M411	10	0	0	0	0	1	0	>512	8	≤0.25	≤0.015	≤2	0.06	≤2	≤4	≤8	≤0.25	≤0.25	≤0.25	≤1	≤1	2
M214	14	1	0	0	0	0	0	>512	8	≤0.25	≤0.015	≤2	≤0.03	≤2	≤4	≤8	≤0.25	≤0.25	≤0.25	≤1	≤1	2
M280	18	0	0	0	0	0	0	>512	16	≤0.25	≤0.015	≤2	≤0.03	≤2	≤4	≤8	≤0.25	≤0.25	≤0.25	≤1	≤1	2
M242	19	1	0	0	0	0	0	≤8	16	8	≤0.015	32 ^b^	≤0.03	≤2	≤4	≤8	≤0.25	≤0.25	≤0.25	≤1	≤1	2
M273	21	1	1	1	0	0	1	32	≤4	8	≤0.015	32 ^b^	≤0.03	≤2	≤4	≤8	≤0.25	≤0.25	≤0.25	≤1	≤1	≤0.5
M239	22	1	0	0	1	0	0															
M487	23	0	0	0	0	0	0															
M469	28	0	0	0	0	1	0															
M270	28	1	1	1	0	0	1															
M229	30	1	0	0	0	0	0															

^a^ Abbreviations: SMX: sulfamethoxazole; AMI: amikacin; TMP: trimethoprim; CIP: ciprofloxacin; TET: tetracycline; MERO: meropenem; AZI: azithromycin; NAL: nalidixic acid; CHL: chloramphenicol; FOT: cefotaxime; TGC: tigecycline; TAZ: ceftazidime; COL: colistin; AMP: ampicillin; GEN: gentamicin. ^b^ MIC above the epidemiological cut-off recommended by EUCAST for *M. haemolytica* for tetracycline (2 μg/mL).

## Data Availability

All data relevant to the study are included in the article.
